# Investigation of rational drug use behaviors and knowledge levels of older individuals: a cross-sectional study

**DOI:** 10.1590/1806-9282.20230692

**Published:** 2024-01-26

**Authors:** İpek Bakimli, Emel Tuğrul

**Affiliations:** 1 Manisa Merkezefendi State Hospital, Anesthesia Intensive Care Unit – Manisa, Turkey.; 2 Aydın Adnan Menderes University, Nursing Faculty, Department of Fundamentals of Nursing – Aydın, Turkey.

**Keywords:** Rational drug, Older adults, Behavior

## Abstract

**OBJECTIVE:**

The aim of this study was to examine older individuals’ rational drug use behavior, their knowledge of rational drug use, and the factors affecting it.

**METHODS:**

This study was conducted cross-sectionally with 440 patients aged 65 years who received inpatient treatment in internal medicine and surgery clinics between October 2021 and November 2022 using a Rational Drug Use Scale and rational drug use behavior questions.

**RESULTS:**

The findings showed that the mean age of older adults was 72.56±5.84 years, and 51.8% were men. It was determined that 79.1% of the older adults did not check their expiration date before using the medicines, and 85.9% of them retained the remaining medicines after treatment. Results indicated that 77.3% of older adults knew less about rational drug use. Additionally, a significant difference was observed between older adults’ marital status, educational status, possession of outdated drugs at home, self-use of antibiotics without examination, and mean score on the Rational Drug Use Scale (p<0.05).

**CONCLUSION:**

The results showed that the rational drug use knowledge level of older adults was low and that there were differences in the knowledge levels of rational drug use according to certain behaviors and factors.

## INTRODUCTION

Rational drug use (RDU) is a rule that must be followed for patients to take drugs according to their needs, the right dosage, sufficient time intervals, and the least cost to themselves and society^1^. Although more than 50% of drugs are improperly prescribed, distributed, and sold worldwide, 50% of patients cannot take medications correctly, and approximately one-third of the global population cannot access essential drugs^2^.

The proportion of the older population in Turkey was 5.7% in 2000, which is expected to increase to 9.7% in 2021^3^. With aging, the number of chronic diseases increases; therefore, the number of older individuals using drugs is increasing. A study conducted with 300 older individuals reported that 58.3% of the individuals used four or more than four drugs, and 72.7% of these drugs were cardiovascular drugs^4^. Another study of 171 older individuals stated that 42.69% of the older adults used five or more drugs, and 94.5% experienced drug-related side effects^5^.

In one study, the prevalence of irrational drug use (IDU) was 44.2%, and the most commonly used drugs in older adults were analgesics^6^. Another study of 190 older individuals found a positive correlation between IDU use and polypharmacy, polypathology, and hypertension^7^.

Investigating risk factors for drug use in older adults and early intervention with controllable factors can reduce the risk of death^8^. The determination of RDU levels and factors affecting older individuals worldwide and in our country is essential for the health-care system. This study aimed to determine drug use behaviors and knowledge levels in an elderly population. The study will provide scientific contributions to determining the roadmap for RDU and the factors affecting IDU in older individuals. Furthermore, in line with the scientific data obtained at the end of the study, it is predicted that nurses and other health professionals responsible for rational drug management will contribute to taking the necessary precautions regarding the issue in older individuals and the spread of these precautions.

## METHODS

This cross-sectional study was conducted in the internal medicine and surgery clinics of the Aydın Adnan Menderes University Hospital between October 2021 and November 2022.

The number of individuals included in the study sample was determined using the sample calculation method of the unknown universe. Accordingly, the minimum number of individuals to be sampled was calculated based on n=unknown, p=0.50, q=0.50, and t=1.96 (α=0.05) values and found to be 384. Considering the possible loss of cases (approximately 10%) in the study sample, 440 older individuals (228 women and 212 men) were included in the sample using a random sampling method (convenience sampling). Patients with memory and hearing problems and those diagnosed with psychiatric diseases were excluded from the study.

The study data were collected through face-to-face interviews in clinics where the patients received treatment between 08:00 and 17:00 on weekdays. Questions in the form of essential features in which sociodemographic characteristics were questioned, a questionnaire questioning RDU behaviors, and the Rational Drug Use Scale (RDUS) were used to determine the knowledge levels of rational drug use. Data collection lasted for an average of 20–30 min for each participant.

Question form: It comprised 22 questions based on a literature review on rational drug use behaviors in elderly individuals^9–11^.

The RDUS is a 21-item scale used to determine individuals’ RDU knowledge levels^12^. For each situation on the scale, the numbers correspond to statements on the scale; they are evaluated as 0-no, 1-do not know, and 2-yes. As the scores obtained from the scale increased, the knowledge of RDU increased. The cutoff point for the scale was 34, and knowledge of RDU with a score of 35 or above was considered high. Cronbach's alpha coefficient of the scale was 0.789. In this study, Cronbach's alpha of the scale was 0.495.

The SPSS Windows software version 25.0 was used to evaluate the data. The data analysis used the percentage distribution and descriptive statistics to define sociodemographic characteristics. The data conformity to the normal distribution was checked using the Kolmogorov-Smirnov test of normality (RDUS: K-S:0.120, p=0.000). Since the data did not show a normal distribution, the Mann-Whitney U, Kruskal-Wallis, and Bonferroni tests were used. Statistical significance was set at p<0.05.

## RESULTS

The mean age of 440 older people included in the study was 72.56±5.84 years; 51.8% were men, 23.9% were hospitalized in internal medicine clinics, 75.7% were married, 54.1% were primary school graduates, and 48.6% were stay-at-home parents. It was found that 91.6% had an income equal to their expenses, and 75.7% lived with their spouses. Among the older participants, 78.6% had a chronic disease, 45.2% had hypertension, 26.6% had diabetes mellitus, 13.6% had heart disease, 5.2% had chronic kidney failure, 16.8% had chronic obstructive pulmonary disease, 8.6% had asthma or bronchitis, 5.0% had epilepsy, and 10.9% had prostate disease. It was determined that 89.5% of the participants were 1 km or closer to their health institution; 85.9% used regular drugs; 29.4% used three drugs; 21.7% used antihypertensive drugs; and 12.6% used diabetic drugs. Behaviors of older adults regarding rational drug use are presented in Table 1.

**Table 1:** Behaviors of older adults regarding rational drug use (n=440).

Behaviors		n	%
Using the drug recommended by a neighbor or a relative			
	Yes	32	7.3
	No	408	92.7
Persons who received information on how to use regularly used drugs			
	Physician	366	83.2
	Pharmacist	74	16.8
	Nurse	0	0
Getting information about the side effects of the drugs used			
	Yes	5	1.1
	No	435	98.9
Reading the leaflet before using the medicines			
	Yes	47	10.7
	No	393	89.3
Status of remaining drugs after treatment			
	Save it in case you need it again	378	85.9
	Giving to those who need it	8	1.8
	Castaway	38	8.6
	Other	2	0.5
	Take to pharmacy	14	3.2
Paying attention to the storage conditions of medicines at home			
	Yes	395	89.8
	No	45	10.2
Storing medicines in their own box			
	Yes	409	93.0
	No	31	7.0
The medicine storage place at home			
	A special drawer	251	57.0
	A special locker	71	16.1
	Medicine cabinet	14	3.2
	Freezer	92	20.9
	Other	12	2.7
Buying medicine from the pharmacy without a physician's examination			
	Yes	101	23.0
	No	339	77.0
Experiencing side effects of any drug used			
	Yes	32	7.3
	No	408	92.7
What to do in case of side effects (n=32)			
	Contacting a physician	30	93.75
	Contacting a pharmacist	2	6.25
Taking the medication with television or media promotion without consulting a physician			
	Yes	3	0.7
	No	437	99.3

Distribution of Rational Drug Use Scale mean and median scores and knowledge levels of older adults are presented in Table 2. There was a significant difference (p<0.05) between marital status, educational level, control of the expiration date of the drugs, possession of expired drugs at home, self-use of antibiotics without examination, and RDUS score averages of older adults. Additionally, the RDUS score average of older adults who did not control the expiration date of the drugs was lower than the average score of older adults who did. It was observed that the mean RDUS score of older adults who did not use antibiotics themselves was higher than that of those who used them (Table 3).

**Table 2:** Distribution of Rational Drug Use Scale mean and median scores and knowledge levels of older adults (n=440).

Scale		mean±SD	Median (QS)	min–max
RDUS		32.52±2.93	32.00 (3.00)	21.00–41.00
			n	%
RDU level of knowledge				
	Low level of knowledge (≤34 points)		340	77.3
	High level of knowledge (>35 points)		100	22.7

QS: quarterly span; SD: standard deviation; RDUS: Rational Drug Use Scale.

**Table 3:** Comparison of some descriptive characteristics and rational drug use behaviors of older adults and Rational Drug Use Scale mean or median scores (n=440).

Variables		n	mean±SD	Median (QS)	Statistics
Marital status					
	Married	333	32.78±2.67	33.00 (3.00)	z: −2.573
	Single	107	31.69±3.51	32.00 (4.00)	p: 0.010*
Educational levels					
	Illiterate (a)	18	29.22±3.81	28.50 (3.75)	
	Literate (b)	21	30.09±4.14	32.00 (6.50)	x²: 26.823
	Primary school (c)	238	32.68±2.59	33.00 (3.00)	p: 0.000*
	Middle school (d)	136	32.91±2.54	33.00 (3.75)	a<c=d=e**
	High school/university (e)	27	33.22±3.75	33.00 (6.00)	
Remembering the time and dose while using the drug					
	Yes	396	32.70±2.66	33.00 (3.00)	z: −2.328
	No	44	30.90±4.46	32.00 (7.75)	p: 0.020*
Checking the expiration date before using medication					
	Yes	92	33.46±3.55	33.00(4.00)	z: −2.932
	No	348	32.27±2.70	32.00(3.00)	p: 0.003*
Presence of expired medication in the home					
	Yes (a)	12	33.91±3.20	33.00(4.75)	x²: 9.433
	No (b)	104	33.26±3.40	33.00(4.00)	p: 0.009*
	I don't know (c)	324	32.23±2.71	32.00(3.00)	b>c***
Using antibiotics without examination					
	Yes	10	28.50±2.95	28.00(4.50)	z: −3.804
	No	430	32.61±2.87	33.00(3.00)	p: 0.000*

* p<0.05

*** p-value obtained as a result of Bonferroni correction p<0.0167, QS: quadrants span; SD: standard deviation; z: Mann-Whitney U test; x^2^: Kruskal-Wallis test.

## DISCUSSION

Most older participants in our study used drugs regularly, and 29.4% used three drugs. The most commonly used drugs are antihypertensive, diabetic, and heart drugs. In a health center study, it was found that older adults mostly used drugs from the cardiovascular (53.5%) and endocrine (9.5%) systems^13^. Chronic diseases that are frequently observed in older adults have led to the use of many drugs. A study on older patients discharged from the hospital reported that 92.1% of older adults were prescribed between 3 and 19 drugs for use after discharge^14^. The use of many drugs by older adults indicates that they are a special group that should be given more importance in RDU.

In our study, most older patients took medication after consulting a physician when sick. In a study conducted by Yılmaz et al.^15^ on older individuals, it was reported that 54.1% of the participants applied to a physician when sick, and 29.1% took medication by applying to a pharmacist. This result shows that older individuals try to solve their health problems by getting help from a health institution as they should; this can be considered positive behavior. Almost all older adults did not use antibiotics independently without an examination because of flu, cold, and flu complaints. Older adults commonly use painkillers, stomach protectors, cold drugs, and vitamins without a prescription^5,15–17^. The absence of over-the-counter antibiotic sales in our country has created a favorable situation by limiting the uncontrolled use of antibiotics. It was determined that a tiny proportion of elderly individuals used drugs recommended by their neighbors or relatives. Studies conducted in our country have reported that the rate of drug use based on the advice of neighbors, family, and friends is low among older adults^5,16,17^.

In this study, most older individuals did not check their expiration date before drug use. Most participants kept the drugs in their boxes and paid attention to their storage conditions. In the literature, most older adults who know the storage conditions of drugs^18,19^ store drugs at room temperature, and individuals between the ages of 60 and 65 years prefer refrigerators^16^.

In our study, most older adults (91.4%) used prescribed drugs. However, the literature has stated that older adults use their drugs for different durations or in different ways than desired^8,15,18–20^.

RDU knowledge levels were evaluated using the scale used in the study. Accordingly, it was determined that participants’ RDU knowledge level was low. In studies conducted using the same scale, different results were obtained regarding knowledge levels in older individuals^21–24^. The scale included topics such as the method of using drugs, their side effects, the use of multiple drugs, the duration of treatment, and what to do in case of undesirable effects. Accordingly, it can be said that due to the lack of knowledge among older adults about drugs, these issues should be addressed. It is estimated that the emergence of progressive memory problems with increased multimorbidity and multiple drug use in aging individuals may affect their RDU knowledge level. It is predicted that the knowledge level regarding RDU can be increased by educating older adults.

In our study, among the factors affecting the RDU knowledge level of older individuals, increasing the education level of individuals and being married stood out as factors that positively affected the average score of rational drug use knowledge level. The mean score of the illiterate elderly was lower than that of those with other educational levels. Education levels can affect individuals’ reading comprehension levels. Therefore, it can be seen that these individuals have higher RDU knowledge levels. In the studies reviewed in the literature, it was observed that individuals’ RDU behaviors and knowledge levels were affected by many variables. Hence, generalizations could not be made^16,25^.

## CONCLUSION

In our study, although most of the RDU behavioral data of older people were positive, their knowledge level of RDU was low. The observed situation can be attributed to older individuals maintaining their established behaviors, which align with their long-standing habits. Furthermore, the decline in learning skills and memory abilities that accompany aging significantly affects their knowledge levels.

Continuous training of individuals, families, and health professionals in light of the information in our study is recommended. Governments should initiate activities to improve the level of knowledge of RDU, create a standardized RDU chain in health institutions, and prepare drug prospectuses more simply and understandably.
